# Mobile Powerhouses: Mitochondria Transfer via Tunnelling Nanotubes in Brain Health and Neurodegenerative Diseases

**DOI:** 10.1111/ejn.70463

**Published:** 2026-03-19

**Authors:** Anna Henrich, Hannah Scheiblich

**Affiliations:** ^1^ Max‐Planck‐Institute for Biology of Ageing Cologne Germany; ^2^ Cluster of Excellence Cellular Stress Response in Aging‐Associated Diseases (CECAD) University of Cologne Cologne Germany

**Keywords:** actin dynamics, cell–cell connectivity, cytoskeletal remodelling, intercellular communication, Miro1/2

## Abstract

Mitochondria are central regulators of cellular metabolism, calcium homeostasis and survival. Owing to the brain's exceptional energy demand, mitochondrial dysfunction is tightly linked to neurodegenerative and neuroinflammatory disorders. Recent evidence challenges the traditional view of mitochondria as strictly cell‐autonomous organelles, revealing that they can be exchanged between cells via intercellular transfer by extracellular vesicles, gap junctions or tunnelling nanotubes (TNTs) as part of an adaptive mechanism of metabolic support and signalling. Among the pathways mediating this intercellular exchange, TNTs—thin, actin‐rich cytoplasmic bridges—have emerged as key conduits for mitochondrial transfer in the nervous system. TNTs enable bidirectional exchange of mitochondria between neurons, glia and vascular cells, thereby promoting bioenergetic recovery after injury and modulating immune and inflammatory responses. This review summarizes current evidence for TNT‐mediated mitochondrial transfer in the brain and highlights the underlying molecular mechanisms that coordinate mitochondrial movement, including cytoskeletal dynamics, mitochondrial trafficking machinery and stress‐induced signalling cascades. While mitochondrial donation can restore metabolic balance and promote neuroprotection, it may also facilitate the spread of pathological proteins, contributing to disease progression. Understanding the underlying molecular mechanism of TNT‐mediated mitochondrial transfer provides a new framework for exploring metabolic communication and cellular resilience in the brain. By emphasizing emerging conceptual and mechanistic insights, we outline how advancing this field could pave the way for the development of innovative therapeutic strategies for neurodegenerative and neuroinflammatory disorders.

## Graphical abstract

Within the brain microenvironment, mitochondria are preferentially transferred from glial cells to metabolically stressed neurons via tunnelling nanotubes (TNTs). This intercellular exchange restores bioenergetic homeostasis and contributes to mitochondrial quality control through transmitophagy. In contrast, malignant cells can hijack TNT‐mediated mitochondrial transfer to fuel metabolic reprogramming, promote tumour growth and drive chemoresistance.

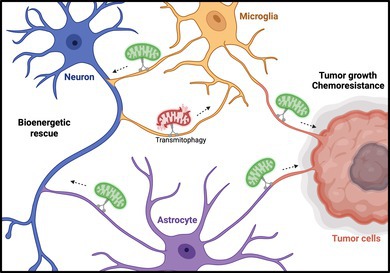

**Abbreviations**
AD: Alzheimer's disease
ATP: adenosintriphosphate
A*β*
: amyloid‐*β*
BBB: blood–brain barrier
BMSC: bone marrow mesenchymal stem cells
Ca^2+^
: calcium
CNS: central nervous system
CX43: connexin‐43
DNTs: dendritic nanotubes
EV: extracellular vesicle
H_2_O_2_
: hydrogen peroxide
IP‐TNTs: interpericyte tunnelling nanotubes
mHTT: mutant Huntingtin
Miro1/2: mitochondrial Rho GTPase 1/2
mRNA: messenger ribonucleic acid
MSC: mesenchymal stem cell
mtDNA: mitochondrial DNA
NSC: neuronal stem cell
PD: Parkinson's disease
ROS: reactive oxygen species
TNFAIP2: M‐Sec (Tumor Necrosis Factor‐*α*‐Induces Protein 2)
TNT: tunnelling nanotube
TUNTs: tunnelling nanotubular networks
*α*‐syn: *α*‐synuclein

## 1 Mitochondrial Vulnerability and Metabolic Cooperation in the Brain

### 1.1 Metabolic Demands of Neurons

The human brain accounts for only about 2% of total body mass, yet it consumes approximately 20% of the body's oxygen at rest (Attwell and Laughlin 2001; Howarth et al. 2012). This striking metabolic imbalance reflects the immense metabolic demands of approximately 86 billion neurons. Each neuron exhibits a highly specialized morphology, comprised of a soma, dendrites and an axon that can extend up to 1 m in humans. Maintaining these complex structures requires a continuous and tightly regulated supply of ATP, making neurons particularly vulnerable to energy deficits. Remarkably, axons alone consume about 55% of neuronal energy, underscoring the high metabolic cost of sustaining long‐range projections (Harris et al. 2012; Rangaraju et al. 2014). This energy supports essential neuronal functions, including synaptic transmission, vesicle cycling and calcium (Ca^2+^) buffering (MacAskill et al. 2010; Schwarz 2013; Lee et al. 2018). These structural and functional features underscore the intimate coupling between neuronal architecture and energetic requirements.

Because of these demands, neurons rely predominantly on oxidative phosphorylation (OXPHOS) rather than glycolysis (Koopman et al. 2013; Zheng et al. 2016). To match local energy needs, mitochondria undergo continuous transport and positioning, accumulating at synapses and nodes of Ranvier (Fabricius et al. 1993; Shepherd and Harris 1998; Hollenbeck and Saxton 2005; Sheng 2014) that require high energy availability.

Beyond ATP production, mitochondria function as signalling hubs (Monzel et al. 2023), releasing mitochondrial DNA (mtDNA), reactive oxygen species (mtROS) and mitochondria‐derived vesicles that regulate inflammation and cell‐fate pathways. Quality control processes, including fusion and fission, mitophagy, biogenesis and proteostasis, are essential for maintaining functional mitochondrial pools, particularly in distal axons with limited turnover capacity (Misgeld and Schwarz 2017). Accordingly, spatial mitochondria distribution emerges as a key regulator of synaptic and axonal homeostasis as well as neuronal performance overall.

### 1.2 Mitochondrial Dysfunction in Neurodegeneration

Given this centrality, mitochondrial impairment contributes broadly to neurological diseases. Mitochondrial disorders are among the most common inherited neurological conditions, and dysfunction is a defining feature of major neurodegenerative diseases (Klemmensen et al. 2024). In Alzheimer's disease (AD), mitochondria display abnormal fusion‐fission dynamics (Wang et al. 2008), altered distribution within pyramidal neurons (Pickett et al. 2018), pronounced morphological changes in *postmortem* brain tissue (Saraiva et al. 1985), impaired biogenesis, defective mitophagy and disrupted proteostasis (Nixon and Yang 2011; Ye et al. 2015). Amyloid‐*β* (A*β*), whose accumulation in the brain is a hallmark of AD, directly interacts with mitochondrial proteins such as alcohol dehydrogenase and cyclophilin D, reducing cytochrome c oxidase activity and disturbing intracellular Ca^2+^ homeostasis (Swerdlow 2018). In Parkinson's disease (PD), mitochondrial impairment is prominently linked to reduced complex I activity, increased oxidative stress, ATP deficiency (Moon and Paek 2015; Henrich et al. 2023; Hattori and Sato 2024) and abnormal mitochondrial morphology along with excessive fragmentation (Knott and Bossy‐Wetzel 2008). *α*‐synuclein (*α*‐syn), the major constituent of Lewy bodies and a pathological hallmark of PD, exacerbates these abnormalities by binding outer membrane lipids, thereby promoting fission (Nakamura et al. 2011; Ryan et al. 2018) and disrupting anterograde axonal transport (Prots et al. 2013; Prots et al. 2018). This, in turn, limits the delivery of functional mitochondria to synaptic terminals, resulting in energy deficits that impair neuronal communication. Mutations in the PINK1‐Parkin pathway, associated with familial PD further impair mitophagy, comprising the clearance of damaged mitochondria, highlighting genetically defined routes to mitochondrial failure (Alqahtani et al. 2023).

### 1.3 Intercellular Mitochondrial Transfer

Given the central role of mitochondrial dysfunction in neurodegeneration, increasing attention has turned to compensatory mechanisms that sustain neuronal viability. Although mitochondria were long believed to arise exclusively through vertical inheritance during cell division or local biogenesis (Mishra and Chan 2014), accumulating evidence now demonstrates that they can also be transferred horizontally between cells under both physiological and pathological conditions (Borcherding and Brestoff 2023). The first evidence for such intercellular mitochondrial transfer was reported in 2006, when adenocarcinoma (A549) cells lacking mtDNA (*ρ* ^0^ cells) regained mitochondrial respiration and proliferative capacity after co‐culture with bone marrow stem cells (BMSCs) (Spees et al. 2006). Since then, intercellular mitochondrial transfer has emerged as a fundamental mechanism for maintaining tissue integrity by buffering metabolic stress, restoring bioenergetic functions and promoting recovery after injury in recipient cells (Khattar et al. 2022).

**FIGURE 1:**
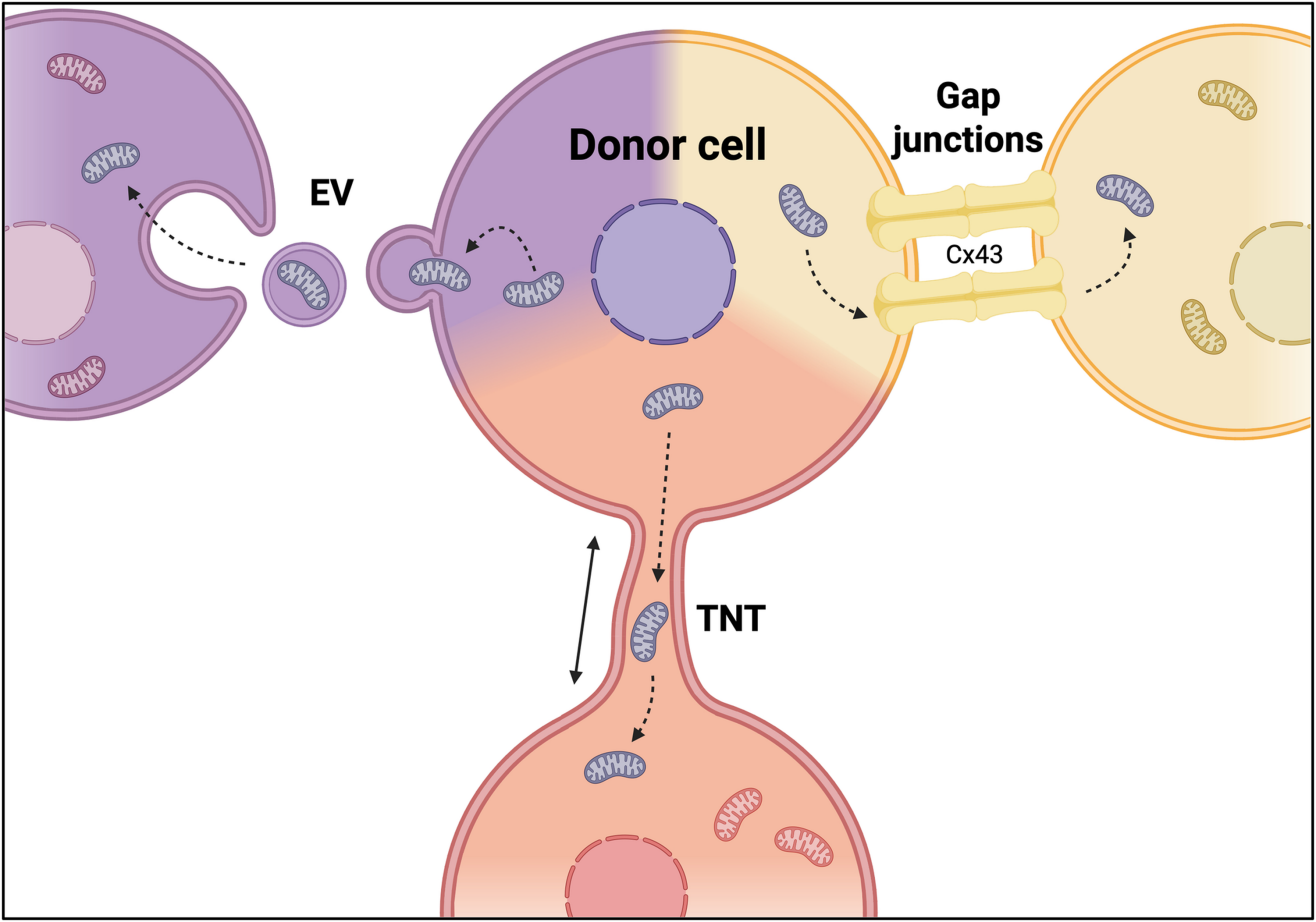
Intercellular transfer of mitochondria via multiple routes. Donor cells can transfer mitochondria to recipient cells through extracellular vesicles (EVs) (D'Souza et al. 2021; Peruzzotti‐Jametti et al. 2021; Yu et al. 2022; Dave, Stolz, Venna, et al. 2023; Wang et al. 2025), tunnelling nanotubes (TNTs) (Rustom et al. 2004; Sartori‐Rupp et al. 2019; Scheiblich et al. 2021; Saha et al. 2022; Chen, Xiao, and Li 2024; Scheiblich et al. 2024) or gap junctions (Norris 2021; Ren et al. 2022; Cetin‐Ferra et al. 2023). EVs mediate the release and uptake of mitochondria‐containing vesicles. TNTs form direct cytoplasmic connections, allowing organelle trafficking between cells. Gap junctions, including those containing Cx43, provide additional conduits for mitochondrial exchange.

Several pathways have been proposed to facilitate this intercellular mitochondrial transfer, ranging from extracellular vesicles (EVs) to transient cell‐to‐cell contacts such as connexin‐43‐mediated gap junctions and tunnelling nanotubes (TNTs) (Torralba et al. 2016) (Figure 1).

EV‐mediated mitochondrial transfer has received considerable attention, particularly in the context of stem cell research. Functionally intact mitochondria—characterized by preserved ultrastructure, membrane potential, and respiratory capacity—can be packed into EVs and delivered to recipient cells (Figure 1). For example, neuronal stem cells have been shown to transfer mitochondria that enhance survival and bioenergetic recovery in recipient cells (Peruzzotti‐Jametti et al. 2021). Similar protective effects have been reported in other cellular contexts, including mitochondrial transfer to endothelial cells in ischemic brain regions, restoring metabolic function (Dave, Stolz, and Manickam 2023; D'Souza et al. 2021), as well as transfer between BMSCs and chondrocytes (Yu et al. 2022). However, EV‐mediated mitochondrial transfer faces quantitative limitations as only approximately 25% of EVs released from MSCs contain mitochondria (Morrison et al. 2017), which poses challenges for therapeutic translation. Recent work has shown that EV‐mitochondria release is regulated by a CD38/IP3R/Ca^2+^‐dependent pathway, and genetic enhancement of this signalling axis can generate ‘super‐donor’ MSCs with markedly increased mitochondrial EV yield and therapeutic efficacy in mitochondrial disease models (Wang et al. 2025). Consequently, current strategies aim to increase mitochondrial yield, for example by enhancing mitochondrial biogenesis in donor cells (Dave, Stolz, and Manickam 2023; Dave, Stolz, Venna, et al. 2023; Wang et al. 2025). In parallel, MSCs have been shown to export damaged mitochondria via arrestin‐domain‐containing protein 1‐mediated microvesicles to macrophages, thereby engaging an intercellular mitophagy‐like pathway (Phinney et al. 2015).

Connexin‐43 (Cx43) mediated gap junctions represent another potential pathway for mitochondrial exchange within the central nervous system (CNS) (Figure 1). Structural evidence from electron microscopy and immunogold labelling suggest close association of mitochondria with gap junctional interfaces (Norris 2021; Cetin‐Ferra et al. 2023). In models of traumatic brain injury, mitochondrial transfer between astrocytes and neurons increase alongside Cx43 upregulation, whereas Cx43 inhibition reduces transfer efficiency (Ren et al. 2022). Mechanistically, gap junctions may facilitate organelle passage under conditions of membrane remodelling or transient pore expansion, although whether intact mitochondria traverse canonical junctional channels or rely on junction‐associated membrane dynamics remains unresolved (Cetin‐Ferra et al. 2023). This suggests that gap junction‐mediated transfer may be context‐dependent and potentially restricted by structural constraints. Therefore, its capacity to accommodate intact organelles remains under debate.

Compared to EVs and gap junctions, TNTs differ fundamentally by forming actin‐based, cytoplasmically continuous bridges (Figure 1) that allow active, motor‐driven transport of mitochondria along defined cytoskeletal tracks. This architecture enables directional, regulated long‐distance organelle trafficking between donor and recipient cells under conditions of metabolic stress or injury. However, despite this progress, major gaps remain regarding the regulation, cell‐type specificity and consequences of TNT‐mediated mitochondrial transfer in the CNS.

This review summarizes current knowledge on TNT‐based mitochondrial exchange in the brain, examining underlying mechanisms, participating cell types and implications for both physiological function and disease, while identifying key priorities for future research.

## 2 Tunnelling Nanotubes: Structure, Diversity and Cellular Distribution

### 2.1 Definition and Morphology

TNTs, first described in 2004 in neuronal pheochromocytoma (PC12) cells (Rustom et al. 2004), are thin, F‐actin‐based membranous protrusions that establish direct cytoplasmic continuity between cells. Through these transient connections, cells can exchange diverse cargos, including ions, proteins, vesicles and whole organelles such as mitochondria (Rustom et al. 2004; Watkins and Salter 2005; Astanina et al. 2015). This enables rapid and regulated long‐distance intercellular communication. Unlike other protrusive structures such as filopodia and cytonemers, TNTs form non‐adherent, free‐floating bridges that span over the substratum in 2D cultures (Cordero Cervantes and Zurzolo 2021).

TNTs typically range from 50 to 700 nm in diameter and can extend across tens of hundreds of micrometres, with both open‐ and close‐ended architectures reported (Cordero Cervantes and Zurzolo 2021). This structural heterogeneity suggests functional diversity, although the molecular mechanisms governing TNT initiation, stabilization, and cargo specification remain incompletely understood (Dagar et al. 2021). In neuronal cell lines such as CAD and SH‐SY5Y cells, multiple TNTs can fuse into bundled structures stabilized by N‐cadherin‐positive junctions, indicating an additional level of architectural coordination and potentially selective signalling regulation (Sartori‐Rupp et al. 2019).

Importantly, TNTs are not restricted to a particular cell lineage or tissue context. They have been observed in a wide variety of cells, including neurons, astrocytes, microglia (English et al. 2020; Scheiblich et al. 2021; Raghavan et al. 2024; Scheiblich et al. 2024; Xi et al. 2024), glioma and other tumour cells (Sahu et al. 2018; Jing et al. 2024; Cangkrama et al. 2025; Solimando et al. 2025), myeloid cells (Dufrançais et al. 2021), mesenchymal stem cells (MSCs) (Baldwin et al. 2024; Huang et al. 2024), pericytes (Alarcon‐Martinez et al. 2020), immune cells (Zhu et al. 2021) and the developing mammalian heart (Miao et al. 2025). This wide distribution highlights their role as versatile conduits operating across both physiological and pathological states.

Within the CNS, TNTs have emerged as critical mediators of neuron–glia communication (Loria et al. 2017; Chakraborty et al. 2023; Scheiblich et al. 2024). They are increasingly implicated in metabolic coupling (Brunialti et al. 2025; Westi et al. 2025), immune signalling and the intercellular exchange of mitochondria and other organelles (Rustom et al. 2004; Abounit, Bousset, et al. 2016; English et al. 2020; Scheiblich et al. 2024). Notably, current evidence suggests that TNTs originate more frequently from glial rather than from neuronal cells, a phenomenon that parallels findings in tumour networks and may reflect a stress‐responsive or metabolism‐dependent initiation mechanism (Du et al. 2021; Chakraborty et al. 2023; Scheiblich et al. 2024).

Although major advances have been made in characterizing TNT distribution, ultrastructure and cargo selectivity, the molecular mechanisms behind their biogenesis remain incompletely resolved. Understanding the cues that trigger TNT formation—particularly under stress, injury or high metabolic demand—represents a major unresolved question with direct implications for their roles in neuroprotection, neuroinflammation, and mitochondrial transfer.

### 2.2 Molecular Regulation of TNT Biogenesis

**FIGURE 2:**
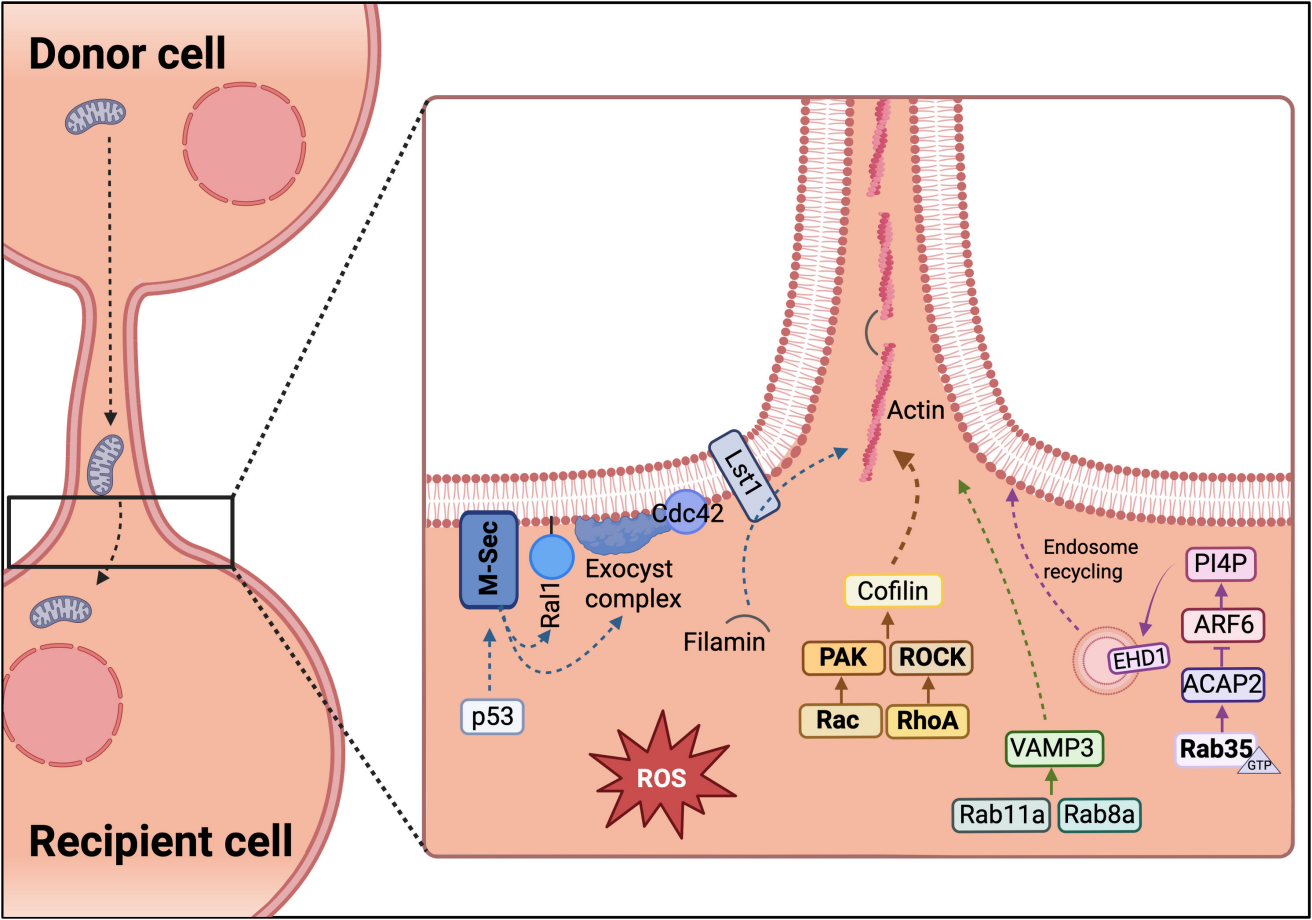
Molecular regulation of TNT biogenesis. TNT formation is driven by coordinated cytoskeletal remodelling, membrane trafficking and stress signalling. Actin polymerization is controlled by small GTPases (Zhu et al. 2016; Zhu et al. 2018; Bhat et al. 2020) and their effectors, while M‐Sec signalling via the exocyst complex stabilizes filaments and supports membrane protrusion (Hase et al. 2009; Kimura et al. 2016; Hanna et al. 2019; Barutta et al. 2021; Che et al. 2025). Endosomal recycling and vesicular trafficking further promote TNT elongation. Reactive oxygen species and stress‐responsive proteins can also induce TNT formation.

Recent research reveals that TNT biogenesis is a tightly regulated, context‐dependent process that integrates cytoskeletal remodelling, membrane trafficking and stress‐response signalling pathways. Dissecting the molecular regulators of TNT formation (Figure 2) is crucial for understanding how these structures are initiated, stabilized and functionally adapted under physiological versus pathological states.

Two principal models have been proposed for TNT biogenesis. In the *cellular dislocation mechanism*, two cells initially establish close membrane contact or transient membrane fusion and subsequently migrate apart while maintaining a thin membranous bridge (Reichert et al. 2016). Rather than arising from active protrusions, these TNTs represent membrane tubes that are mechanically stretched and stabilized during cell separation. Live‐cell imaging has provided evidence for this process in multiple cell types, such as activated T‐cells (Sowinski et al. 2008), PC12 cells (Bukoreshtliev et al. 2009), macrophages (Önfelt et al. 2006), SH‐SY5Y and primary brain pericytes (Dieriks et al. 2017), and hematopoietic progenitors (Reichert et al. 2016). Importantly, adequate cell–cell contact appears to be a prerequisite: for example, T cells form TNTs only after several minutes of sustained contact (> 4 min), indicating that transient membrane interactions are insufficient (Sowinski et al. 2008). Emerging evidence further suggests that cellular dislocation‐driven TNT formation is modulated by the microenvironment. Alterations in cell adhesion properties, mechanical tension and extracellular matrix stiffness influence the likelihood that separated cells retain membranous connections (Mahadik and Patwardhan 2023). In addition, biochemical cues such as exosomal thrombospondin‐1 have been implicated in promoting TNT formation by modifying adhesion dynamics and membrane remodelling (Mahadik and Patwardhan 2023). Together, these findings indicate that TNT biogenesis via cellular dislocation is not purely mechanical but regulated by integrated biophysical and biochemical signals.

In contrast to this mechanically driven mechanism, the *actin‐driven model* involves the active extension of a filopodia‐like protrusion from one cell towards another, followed by membrane fusion and establishment of cytoplasmic continuity (Rustom et al. 2004; Castro et al. 2005; Sherer and Mothes 2008; Hase et al. 2009; Wang et al. 2010; Cordero Cervantes and Zurzolo 2021). Among the best‐characterized regulators of the actin‐driven pathway is TNFAIP2 (M‐Sec), whose overexpression promotes TNT formation in several cell types including hippocampal astrocytes, macrophages, podocytes and MSCs (Kimura et al. 2016; Hanna et al. 2019; Barutta et al. 2021; Che et al. 2025). M‐Sec interacts with the small GTPase RalA, a central regulator of actin remodelling (Chamberlain et al. 2021), and with components of the exocyst complex (Hase et al. 2009) (Figure 2). Within this complex, Lst1 stabilizes RalA‐exocyst interactions and recruits the actin crosslinker filamin (Schiller et al. 2013), while Cdc42, although essential for filopodia initiation, appears to participate primarily in TNT elongation rather than nucleation (Hase et al. 2009). Additional M‐Sec interactors, including ERp29, which facilitates M‐Sec localization and stabilization at the plasma membrane, and nucleolin, which regulates cortical actin dynamics by stabilizing specific mRNAs (Pergu et al. 2019), further refine this regulatory network.

Beyond the M‐Sec axis, Rab GTPases including Rab35, Rab11a, and Rab8a contribute to TNT biogenesis and cargo trafficking (Zhu et al. 2016; Zhu et al. 2018; Bhat et al. 2020) (Figure 2). In neuronal CAD cells, Rab35 promotes TNT formation via ACAP and ARF‐GDP signalling (Bhat et al. 2020). In contrast, in primary neuron–microglia co‐cultures, TNT biogenesis depends on the Rac‐PAK pathway, a key mediator of actin polymerization and protrusion dynamics (Figure 2). Inhibition of either Rac or PAK substantially reduces TNT formation between the two cell types (Scheiblich et al. 2024). Consistent with these findings, earlier work demonstrated a role for RhoA and its downstream effector ROCK in microglial TNT formation. Inhibition of ROCK by Y‐27632 increases the number of TNTs connecting microglia and enhances the transfer of cellular protein aggregates through TNTs, likely reflecting prolonged structural stability (Scheiblich et al. 2021; Yuan et al. 2024). Pharmacological studies further highlight the central role of actin dynamics in TNT biogenesis. Low‐dose cytochalasin D, which inhibits cofilin‐mediated actin turnover, effectively suppresses TNT formation between neurons and microglia while leaving the phagocytic capacity of microglia intact (Scheiblich et al. 2024).

TNT formation is strongly induced by stress‐related cues, including oxidative stress stimuli such as hydrogen peroxide (H_2_O_2_) (Wang et al. 2011), mitochondrial dysfunction and the presence of misfolded proteins (Wang et al. 2011; Zhang et al. 2021; Tarasiuk and Scuteri 2022; Scheiblich et al. 2024). These stressors converge on pathways, regulating lysosomal stress, ROS production and redox homeostasis, conditions frequently observed in neurodegenerative settings. In astrocytes, oxidative stress activates p53, which stimulates the PI3K‐AKT‐mTOR axis to upregulate M‐Sec expression and promote actin polymerization, thereby inducing TNT formation (Wang et al. 2011; Lin et al. 2024). Beyond these general stress responses, TNT formation has been consistently observed in response to defined pathophysiological stressors relevant to neurological disease. In particular, ischemic injury and ischemia/reperfusion‐like conditions robustly induce TNT formation in multiple neuronal and vascular cell types (Wang et al. 2011; Pisani et al. 2022; Capobianco et al. 2024). Oxygen–glucose deprivation, a widely used in vitro model of cerebral ischemia, increases the number and length of TNTs in neurons, glia cells and blood–brain barrier (BBB) cells, concomitant with enhanced intercellular mitochondrial transfer (Pisani et al. 2022; Capobianco et al. 2024; Xi et al. 2024). Importantly, TNT formation under these conditions is highly context‐ and cell‐type dependent, with astrocytes and pericytes acting as prominent mitochondrial donors, while neurons or endothelial cells often function as metabolically compromised recipients. Moreover, studies in cancer and injury models indicate that therapeutic or toxic stressors, including irradiation and pharmacological treatments, induce TNT formation through signalling pathways involving PI3K/AKT, MAPK, and actin‐remodelling proteins such as Eps8 and cofilin, mechanisms that are likely relevant to stressed neural tissues (Kretschmer et al. 2019; Cole et al. 2021; Lin et al. 2024). Together, these findings show that TNT formation is controlled by multiple factors, combining the coordinated interplay between the actin cytoskeletal machinery, membrane trafficking complexes, and stress‐adaptative signalling pathways (Figure 2). Rather than representing a constitutive feature of cellular communication, TNTs appear to be dynamically induced structures that emerge preferentially in metabolically or redox‐challenged environments, potentially as a strategy to combat cellular dysfunction.

### 2.3 Cargo Specificity and Bidirectional Exchange

Far beyond simply connecting neighbouring cells, TNTs enable the exchange of an exceptionally broad repertoire of cargo, positioning them as key mediators of intercellular communication, metabolic support and stress adaptation under physiological and pathological conditions.

#### 2.3.1 Physiological Cargoes

Under physiological conditions, TNTs facilitate the transfer of small molecules such as ions, second messengers and metabolic substrates, as well as larger macromolecules including proteins, lipids and nucleic acids (Arkwright et al. 2010; Wang et al. 2010; Rainy et al. 2013; Haimovich et al. 2017; Haimovich et al. 2020). Beyond these soluble components, TNTs also mediate the trafficking of entire organelles, including mitochondria (Wang and Gerdes 2015; Sartori‐Rupp et al. 2019; Saha et al. 2022), lysosomes (Abounit, Bousset, et al. 2016), ribosomes (Martínková et al. 2025), endosomes, peroxisomes and fragments of the endoplasmic reticulum (Davis and Sowinski 2008; Kadiu and Gendelman 2011). In addition, TNTs can transport regulatory and signalling molecules directly involved in cell fate decisions, such as active caspase‐3 (Arkwright et al. 2010), mRNA (Dasgupta et al. 2023; Yoneyama et al. 2025) and Ca^2+^ signalling components (Lock et al. 2016). Through these diverse exchanges, TNTs contribute to metabolic coupling, Ca^2+^ homeostasis, and the coordinated activation of stress‐response pathways between connected cells, thereby influencing survival and adaptation under both physiological and stress conditions.

#### 2.3.2 Pathological Cargoes

However, this same intercellular connectivity can also be exploited under pathological conditions. TNTs have been implicated in the spread of viral particles, including HIV‐1 transmission between T‐cells (Sowinski et al. 2008), intercellular dissemination of prions between neuronal CAD cells (Gousset et al. 2009; Zhu et al. 2015) and neurotoxic protein aggregates such as A*β*, tau, α‐syn, and mutant Huntingtin (mHTT) between neuronal cell lines like CAD, SH‐SY5Y and STHdh^Q7/Q7 cells and primary striatal and cortical neurons (Abounit, Wu, et al. 2016; Dieriks et al. 2017; Zhang et al. 2021; Subramaniam 2022; Scheiblich et al. 2024). A recent study further identified dendritic nanotubes (DNTs), reminiscent of TNTs, as an additional route for intercellular communication among neurons, facilitating the spread of A*β* (Chang et al. 2025). These findings have led to the hypothesis that TNTs and similar structures like DNTs may serve as conduits for the propagation of pathological proteins in neurodegenerative diseases such as AD, PD and Creutzfeldt–Jakob disease.

Beyond neurodegenerative contexts, TNTs are increasingly recognized as mediators of pathological organelle and cytoplasmic transfer in cancer. In glioblastoma, TNTs facilitate the exchange of functional mitochondria and cytoplasmic material from neighbouring cells, supporting metabolic reprogramming, enhancing OXPHOS and promoting survival, thereby contributing directly to chemoresistance (Pinto et al. 2021; Valdebenito et al. 2021; Nakhle et al. 2023). Recent evidence further shows that tumour cells hijack mitochondria from immune cells, impairing antigen presentation and reducing NK and CD8^+^ T‐cell cytotoxicity. In cancer cells, the acquired mitochondria integrate into endogenous networks, induce mtDNA release and activate cGAS/STING‐dependent type I interferon signalling, thereby promoting immune evasion and lymph node metastasis. Inhibition of mitochondrial transfer machinery or downstream signalling reduces metastatic spread, highlighting mitochondrial hijacking as a driver of tumour progression and immune escape (Terasaki et al. 2026).

Together, these findings highlight TNTs as versatile communication highways that, depending on the physiological or pathological context, can either support or disrupt cellular networks. In the nervous system, TNT‐mediated mitochondrial transfer appears to play a particularly important role, helping to buffer metabolic stress, preserve neuronal viability, and maintain overall network integrity. The following section summarizes current evidence for mitochondrial transfer within the brain and its implications in both health and disease.

## 3 Evidence and Mechanism of Mitochondrial Transfer Trough TNTs in the Brain

### 3.1 Cell Type Specificity and Pathological Hijacking of TNT‐Mediated Mitochondrial Trafficking in the CNS

The brain's exceptionally high metabolic demand and cellular complexity, coupled with the limited regenerative capacity of neurons, make mitochondrial homeostasis a central determinant of tissue integrity. Accordingly, accumulating evidence suggests that within the neurovascular unit—encompassing neurons, astrocytes, microglia, endothelial cells and pericytes—cells engage in active mitochondrial exchange via TNTs and related structures under both physiological and pathological conditions. These observations fundamentally shift the view of mitochondria in the brain from being exclusively cell‐autonomous organelles towards shared, dynamically redistributed resources that support circuit resilience.

The first direct evidence for mitochondria within TNTs in the brain in vivo emerged from high‐resolution two‐photon imaging studies demonstrating interpericyte tunnelling nanotubes (IP‐TNTs), which connect adjacent pericytes along the capillary network (Alarcon‐Martinez et al. 2020). Subsequent studies have extended these findings, revealing that IP‐TNTs play a critical role in mitigating microvascular deficits and neurovascular coupling impairments (Kempf et al. 2024). Importantly, work in human BBB co‐culture systems combining brain endothelial cells, pericytes, and astrocytes, as well as in three‐dimensional human BBB assembloids, demonstrated that TNT‐mediated mitochondrial trafficking is not restricted to pericyte‐pericyte connections but also supports functional crosstalk across the tripartite BBB (Pisani et al. 2022). Current evidence supports TNT‐mediated communication predominantly within the cellular architecture of the BBB, rather than the formation of nanotubular conduits that directly traverse the intact barrier into the circulation. In these models, astrocytes form long TNTs with both endothelial cells and pericytes and receive functional mitochondria through these structures, a process that is markedly upregulated under ischemia/reperfusion‐like conditions. Notably, TNT‐mediated mitochondrial transfer from healthy pericytes rescues astrocytes exposed to oxygen–glucose deprivation from apoptosis, an effect that is largely abolished upon pharmacological disruption of TNTs (Pisani et al. 2022). In addition to classical TNTs, high‐resolution scanning electron microscopy studies of brain endothelial cells have described exosome‐derived nanotubular structures, referred to as tunnelling nanotubular networks (TUNTs), which span the paracellular space and form a scaffold‐like network at the apicolateral membrane region (Mentor and Fisher 2022). These TUNTs appear to support inter‐endothelial communication and are proposed to participate in BBB formation and the fine regulation of barrier permeability. Together, these findings establish TNTs and TUNTs as inducible conduits for metabolic cooperation within the BBB. Through coordinated cellular stress responses and the redistribution of organelles such as mitochondria, these nanotubular networks may modulate barrier stability under physiological and pathological conditions. Dysregulation of TNT‐ and TUNT‐mediated communication has therefore been hypothesized to contribute to BBB dysfunction and increased permeability in neurodegenerative or inflammatory disorders.

Astrocytes have also emerged as key donors of mitochondria in the injured brain. Following ischemic stroke, astrocytes form TNT‐like protrusions that transfer functional mitochondria into metabolically compromised neurons, restoring ATP production and supporting neuronal survival (Hayakawa et al. 2016; Xi et al. 2024; Guo et al. 2025; Sun et al. 2026). Related observations at the neurovascular interface show that astrocytes donate mitochondria to endothelial cells and pericytes, particularly in ageing, potentially compensating for vascular energetic decline (Liu et al. 2024; Velmurugan et al. 2024). Notably, analogous glia‐to‐neuron mitochondrial transfer has recently been demonstrated in the peripheral nervous system, where satellite glial cells deliver mitochondria to dorsal root ganglion neurons via Myosin‐10‐dependent TNTs in vivo, and disruption of this process induces neuropathy (Xu et al. 2026). This expanding body of evidence supports the concept of glial cells as central ‘metabolic supporters’ of both neurons and vascular cells, functioning as active organelle reservoirs that stabilize energy supply across neural systems.

Microglia, the brain's resident immune cells, also participate in bidirectional mitochondrial exchange. Under metabolic stress, microglia transfer healthy mitochondria via TNTs to neurons, restoring neuronal mitochondrial membrane potential and reducing ROS production (Scheiblich et al. 2024). Conversely, microglia exchange mitochondria among themselves to limit oxidative stress and modulate inflammatory responses, thereby promoting microglial resilience and preserving tissue homeostasis (Scheiblich et al. 2021). This dual capacity indicates that TNT‐mediated mitochondrial trafficking acts as a key mechanism for both neuroprotection as well as immune regulation in the nervous system.

Neural stem cells (NSCs) which have attracted great interest in regenerative medicine, form TNTs both among themselves and with the neuronal cell line SH‐SY5Y (Capobianco et al. 2024). Similar interactions have been observed between radial glial cells, the progenitors of NSCs, and between brain tumour‐initiating cells and astrocytes (Boyineni et al. 2024). Under hypoxic conditions, these TNTs enable the transfer of mitochondria, thereby attenuating apoptotic processes and restoring the bioelectrical properties of affected neurons (Boyineni et al. 2024; Capobianco et al. 2024). Within the post‐ischemic phase, such TNT‐mediated intercellular communication may thus represent a previously underappreciated mechanism contributing to neuroprotection and functional recovery. Beyond receiving functional mitochondria, neurons can also offload damaged organelles to neighbouring astrocytes and other glia cells through TNTs in a process known as ‘transmitophagy’. In this mechanism, defective mitochondria and mtDNA are shuttled from neurons to astrocytes for degradation (Davis et al. 2014; Lampinen et al. 2022; Solimando et al. 2025). This intercellular quality‐control system not only clears dysfunctional mitochondria but may also activate compensatory responses in astrocytes, inducing increased mitochondrial biogenesis driven by elevated expression of mitochondrial Rho GTPase 1 (Miro1) and TFAM (Gao et al. 2022). Together, these reciprocal exchanges support metabolic balance within the neuro‐glial network and help sustain neuronal survival under challenging conditions.

However, while mitochondrial transfer can be neuroprotective, it is not uniformly beneficial. Cancer cells, including gliomas, exploit TNT‐mediated mitochondrial exchange to enhance tumour growth, survival, metabolic agility and therapy resistance (Dong et al. 2017; Nakhle et al. 2023; Watson et al. 2023; Venkataratnam et al. 2025). Moreover, recent evidence suggests that misfolded proteins such as *α*‐syn hitchhike on transferred mitochondria, enhancing their intercellular spread and potentially accelerating neurodegenerative processes (Valdinocci et al. 2021).

Collectively, these findings highlight mitochondrial transfer as a widespread and dynamic mechanism of intercellular communication in the brain. By enabling cells to share metabolic resources and eliminate damaged organelles, TNTs enhance the resilience and adaptability of neuronal networks, particularly under metabolic or oxidative stress. However, in pathological settings such as cancer or proteinopathy, these same pathways can be exploited to spread dysfunction, inflammation or malignancy.

Despite the accumulating evidence, it is important to recognize that a considerable proportion of studies demonstrating TNT‐mediated mitochondrial transfer rely on in vitro systems and dye‐based labelling approaches such as MitoTracker variants. Although these tools have been instrumental in visualizing intercellular organelle movement, they remain susceptible to artefacts including dye leakage, vesicular transport or nonspecific signal diffusion. Recent studies employing genetically encoded mitochondrial reporters, ultrastructural analyses and in vivo imaging have strengthened the evidence base; however, quantitative data regarding the frequency, persistence and functional integration of transferred mitochondria in the intact brain remain limited. Continued development of orthogonal validation strategies and longitudinal in vivo imaging will therefore be essential to firmly establish the physiological relevance of TNT‐mediated mitochondrial exchange under different conditions.

### 3.2 Mechanisms of Mitochondrial Transport Through TNTs

**FIGURE 3:**
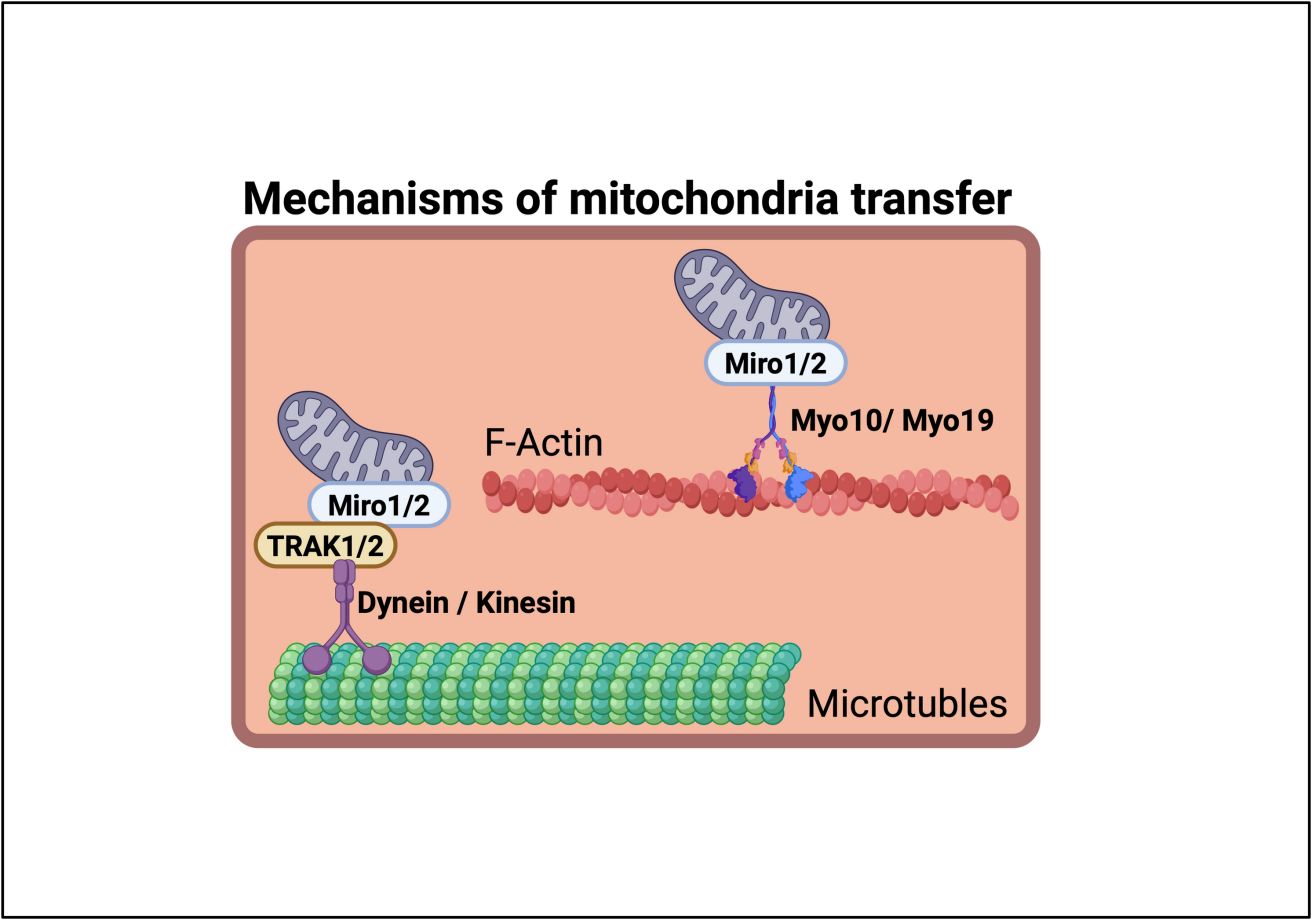
Mechanisms of mitochondrial transport along cytoskeletal tracks. Mitochondria are linked to molecular motors via the outer mitochondrial membrane GTPases Miro1 and Miro2 (Fransson et al. 2006; Devine et al. 2016). In association with adaptor proteins TRAK1/2 (Ahmad et al. 2014), Miro1/2 connect mitochondria to kinesin and dynein motors for bidirectional transport along microtubules. In actin‐rich regions, Miro1/2 associate with Myosin‐10 (Myo10) (Zaninello and Bean 2023; Xu et al. 2026) and Myosin‐19 (Myo19) (Quintero et al. 2009; López‐Doménech et al. 2018; Oeding et al. 2018) to mediate actin‐dependent mitochondrial motility.

Mitochondrial transport through TNTs is a complex, highly regulated multi‐step process that integrates cytoskeletal remodelling with mitochondrial quality control and metabolic stress signalling. While TNTs are predominantly actin‐based structures, their capacity to mobilize and deliver mitochondria depends on a specialized set of adaptor and motor proteins that physically link the organelle to the cytoskeletal tracks (Figure 3).

Central to this process are the outer mitochondrial membrane GTPases Miro1 and Miro2, which function as key adaptors linking mitochondria to molecular motors (Fransson et al. 2006; Devine et al. 2016). In association with the adaptor proteins TRAK1 and TRAK2, Miro proteins recruit kinesin and dynein motors, enabling long‐range bidirectional transport along microtubules (Ahmad et al. 2014) (Figure 3). In parallel, Miro1/2 associate with Myosin‐19, facilitating actin‐based mitochondrial motility that is particularly relevant within TNTs (Figure 3), where microtubules are often sparse or absent (Quintero et al. 2009; López‐Doménech et al. 2018; Oeding et al. 2018). Recent data also implicate Myosin‐10, previously associated with filopodia extension and mitochondrial positioning, as a contributor to TNT formation (Ramirez Perez et al. 2026) and TNT‐mediated mitochondrial movement (Zaninello and Bean 2023; Xu et al. 2026) (Figure 3).

Structurally, Miro1/2 contain two GTPase domains and two EF‐hand motifs that bind Ca^2+^, allowing intracellular Ca^2+^ levels to directly modulate mitochondrial motility. Under elevated intracellular Ca^2+^ levels, Miro1/2 release mitochondria from their cytoskeletal anchors and temporarily immobilize them at subcellular sites where energy demand is highest (Saotome et al. 2008; Wang and Schwarz 2009). This localized anchoring likely plays a critical role during TNT‐mediated transfer, especially near synaptic terminals or injury sites where mitochondrial delivery is functionally required (Hollenbeck and Saxton 2005; Sheng 2014).

Experimental evidence indicates that mitochondrial transport efficiency is directly modulated by Miro1/2 levels in donor cells: Miro1 overexpression enhances, while knockout or silencing markedly reduces intercellular mitochondrial transfer, as shown in a mouse melanoma model (Novak et al. 2025). In regard to transfer to neurons, upregulation of Miro1 also correlates with increased mitochondrial trafficking (English et al. 2020; Sun et al. 2026). Combined, these observations indicate that Miro proteins not only mediate transport but may also contribute to mitochondria localization and presumably the selection of mitochondria destined for export.

Beyond the role in motility, Miro1 contributes to mitochondrial quality control by interacting with the PINK1‐Parkin pathway, which regulates mitophagy. Miro1 serves as a Ca^2+^‐dependent docking platform for Parkin, and Miro1 knockdown markedly diminishes Parkin recruitment to mitochondria, thereby impairing mitophagy (Safiulina et al. 2019). Upon mitochondrial depolarization, PINK1 accumulates on the outer mitochondrial membrane and primes Miro1 for ubiquitination and degradation, halting motility and targeting damaged mitochondria for degradation (Weihofen et al. 2009). This degradation may prevent the transfer of damaged mitochondria to neighbouring cells, providing a quality control checkpoint at the interface between intracellular mitophagy and intercellular organelle exchange.

Together, these mechanisms suggest that TNT‐mediated mitochondrial movement is orchestrated through a multifactorial network that links cytoskeletal architecture, Ca^2+^ homeostasis, metabolic stress responses and mitophagy programs during TNT‐mediated mitochondrial displacement. The dynamic interplay between Miro‐TRAK motor complexes, actin regulators (including M‐Sec and RalA), and mitophagy pathways might ensure that mitochondrial transfer is both spatially targeted and quality‐controlled, enabling cells to adaptively distribute metabolic resources across the neural network.

## 4 Challenges and Future Directions

### 4.1 Methodological Limitations

The discovery that mitochondria can move between cells has fundamentally reshaped our understanding of how the brain might maintain metabolic stability and respond to stress. Rather than functioning as isolated autonomous units, neurons and glia as well as cells from the neurovascular unit appear to operate within a cooperative metabolic network, in which mitochondria are selectively shared, repaired or eliminated through intercellular routes such as TNTs. Through this exchange, cells can restore ATP production, buffer oxidative damage, support recovery after injury and promote cellular functioning and survival. Consequently, intercellular mitochondrial transfer has emerged as a promising therapeutic target in neurodegenerative, ischemic and metabolomic disorders. Yet, despite this rapid conceptual advance, key methodological and mechanistic challenges remain unresolved.

In the intact brain, tracking mitochondrial movement is technically difficult. Widely used mitochondrial dyes (e.g., MitoTracker or Rhodamine derivatives) can dissociate from mitochondria or spread via vesicles, producing false‐positive signals that mimic organelle exchange events in the absence of true transfer (Chen, Li, et al. 2024). Recent studies have therefore emphasized the need for rigorous validation using complementary strategies, including genetically encoded mitochondrial reporters, photo‐convertible fluorophores and correlative ultrastructural imaging, to demonstrate bona fide organelle exchange (Hole et al. 2025). Integrating these approaches with high‐resolution in vivo imaging will be crucial to quantify mitochondrial trafficking dynamics and organelle fate in recipient cells. Moving forward, rigorous evidence will require multiple orthogonal readouts, including functional assays that verify metabolic rescue rather than merely morphological transfer.

### 4.2 Donor‐Recipient Identity: Logics of Directed Transfer

In parallel to these methodological refinements, a new frontier concerns directionality and selectivity, how cells determine who donates and who receives mitochondria. Current evidence suggests that mitochondrial transfer is not random but regulated by relative metabolic status, redox state and cellular stress signals. Astrocytes and microglia typically act as donors of mitochondria (Du et al. 2021; Chakraborty et al. 2023; Scheiblich et al. 2024; Guo et al. 2025), consistent with their greater metabolic plasticity and higher spare respiratory capacity compared to neurons. Conversely, neurons are frequent recipients of mitochondria, particularly under hypoxia, excitotoxicity or oxidative injury (Hayakawa et al. 2016; Capobianco et al. 2024; Scheiblich et al. 2024; Xi et al. 2024; Guo et al. 2025). At the molecular level, Ca^2+^‐sensitive regulators such as Miro1 (Fransson et al. 2006; Devine et al. 2016; López‐Doménech et al. 2018), stress‐responsive signalling cascades (p53, mTOR and NF‐κB) (Wang et al. 2011) and mitophagy‐linked pathways (PINK1‐Parkin) (Weihofen et al. 2009; Safiulina et al. 2019) appear to influence whether mitochondria are retained, degraded or exported. These findings suggest that mitochondrial transfer may represent an extension of the mitochondrial quality control system, with healthy mitochondria preferentially supplied to compromised cells, and damaged mitochondria exported towards healthy cells for degradation (‘transmitophagy’) (Lampinen et al. 2022; Solimando et al. 2025). Looking forward, defining the precise triggers and cellular contexts in which mitochondrial transfer occurs will be critical to understand its selectivity, directionality and physiological relevance.

Nevertheless, several key questions remain unresolved. What molecular and cellular signals determine donor and recipient identity within the nervous system? How frequently does TNT‐mediated transfer occur in the intact brain, and what are its rates and consequences under physiological and pathological in vivo conditions? Moreover, the fate of transferred mitochondria remains poorly understood. Do transferred mitochondria integrate permanently into the host organelle network, undergo partial fusion or are they transient metabolic first aiders? These gaps highlight the need for longitudinal imaging in vivo and improved fate‐mapping strategies capable of tracking transferred mitochondria over days to weeks.

### 4.3 Translational Challenges

Understanding whether and how TNT‐mediated transfer can be selectively and safely modulated in vivo remains a central translational challenge.

TNT‐mediated mitochondrial transfer has emerged as a therapeutically exploitable process, particularly within stem cell and cancer research. MSCs can alleviate mitochondrial dysfunction by donating functional mitochondria to recipient cells via TNTs and EVs, a strategy explored in models of neurodegenerative and other neurological disorders. For example, mitochondrial transfer from MSCs to NSCs confers protection against cisplatin‐induced toxicity in vivo, an effect that is further enhanced by Miro1 overexpression, suggesting increased efficiency of TNT‐mediated transfer (Boukelmoune et al. 2018). Complementary studies in models of PD and AD indicate that mitochondrial transplantation and transfer can reduce neuronal loss, dampen microglial activation (Eo et al. 2024) and mitigate tau and A*β* pathology (Mishra et al. 2023). Therapeutic efforts in this area also focus on investigation of cellular pathways that might regulate TNT formation and function. Induction of autophagy was found to promote TNT length and enhance mitochondria transfer from MSCs (Sadeghsoltani et al. 2024), highlighting regulatory entry points for therapeutic enhancement.

Beyond enhancing endogenous donation, recent studies indicate that mitochondrial transfer capacity can be actively engineered. Downregulation of PINK1‐mediated mitophagy increases intercellular mitochondrial transit by promoting metabolic and structural adaptation in donor cells. In tumour models, such mitochondria were harnessed as TNT‐dependent ‘vehicles’ to deliver photosensitizers, improving tumour penetration and photodynamic efficacy (Peng et al. 2025). These findings underscore that mitochondrial trafficking is not merely compensatory but can be strategically modulated for therapeutic delivery.

In cancer research, TNTs are being targeted through two conceptually distinct strategies. One approach exploits TNTs as conduits for drug delivery, leveraging their capacity for rapid and spatially confined intercellular transport. Structural differences between glioblastoma‐derived TNTs and those of healthy astrocytes have been utilized to selectively deliver nanoparticles into otherwise inaccessible tumour niches (Formicola et al. 2019), and engineered microglia‐like BV2 cells have been employed to shuttle chemotherapeutic agents to glioblastoma cells via TNTs, highlighting the potential of immune cells as transport vectors (Du et al. 2021). Conversely, inhibition of TNT formation aims to prevent pathological mitochondrial exchange and therapy resistance. Blocking TNT‐mediated mitochondria transfer has been shown to limit multidrug resistance in breast cancer cells driven by mitochondria donation from adipose‐derived stem cells (Del Vecchio et al. 2024). Both pharmacological interventions—including inhibitors of actin polymerization, DNA synthesis, and mTOR signalling—and genetic targeting of TNT‐associated proteins are actively being investigated (Chen and Zhao 2024). Despite this progress, major challenges remain. Enhancing mitochondrial delivery to neurons may hold therapeutic promise in neurodegenerative disease, stroke or ageing, but indiscriminate stimulation of TNT formation carries the risks of facilitating tumour progression, pathological aggregate spread, immune activation and potential alterations in mtDNA heteroplasmy. Furthermore, in vivo control over donor‐recipient specificity, transfer frequency and long‐term fate of transferred mitochondria remains poorly defined. Precise modulation strategies, potentially involving cell‐type‐specific targeting of regulators such as Miro1 or M‐Sec, will be required to achieve therapeutic efficacy without unintended consequences.

Collectively, the field is now transitioning from descriptive observations to mechanistic and translational refinement. Establishing quantitative in vivo standards, clarifying donor‐recipient identity/selectivity and resolving the functional fate of transferred mitochondria will be crucial next steps towards harnessing TNT‐mediated transfer as a controlled therapeutic strategy rather than a passive context‐dependent biological phenomenon.

## Author Contributions

**Anna Henrich:** conceptualization, visualization, writing – original draft, writing – review and editing. **Hannah Scheiblich:** conceptualization, visualization, writing – original draft, writing – review and editing.

## Funding

This work was supported by Gemeinnützige Hertie Stiftung P1200007; Gerhard und Ilse Schick Stiftung; Alzheimer Forschung Initiative #23002R; Fritz Thyssen Stiftung Az. 10.25.1.027MN; Deutsche Forschungsgemeinschaft 548919474.

## Conflicts of Interest

The authors declare no conflicts of interest.

## Data Availability

This review article did not generate, collect or analyse any new datasets. All information discussed in this manuscript is derived from previously published studies, which are cited appropriately within the text. Readers are encouraged to consult the original publications cited in this review for access to the underlying datasets, where available.
